# The *Escherichia coli *BarA-UvrY two-component system is a virulence determinant in the urinary tract

**DOI:** 10.1186/1471-2180-6-27

**Published:** 2006-03-10

**Authors:** Henrik Tomenius, Anna-Karin Pernestig, Kristina Jonas, Dimitris Georgellis, Roland Möllby, Staffan Normark, Öjar Melefors

**Affiliations:** 1 Swedish Institute for Infectious Disease Control, SE-17182 Solna, Sweden; 2 Microbiology and Tumorbiology Center, Karolinska Institutet, SE-17177 Stockholm, Sweden; 3 School of Life Sciences, University of Skövde, SE-54128 Skövde, Sweden; 4 Departamento de Genética Molecular, Instituto de Fisiologia Celular, Universidad National Autónoma de México, 04510 México D.F, Mexico

## Abstract

**Background:**

The *Salmonella enterica *BarA-SirA, the *Erwinia carotovora *ExpS-ExpA, the *Vibrio cholerae *BarA-VarA and the *Pseudomonas spp *GacS-GacA all belong to the same orthologous family of two-component systems as the *Escherichia coli *BarA-UvrY. In the first four species it has been demonstrated that disruption of this two-component system leads to a clear reduction in virulence of the bacteria. Our aim was to determine if the *Escherichia coli *BarA-UvrY two-component system is connected with virulence using a monkey cystitis model.

**Results:**

Cystitis was generated in *Macaque fascularis *monkeys by infecting the bladder with a 1:1 mixture of the uropathogenic *Escherichia coli *isolate *DS17 *and a derivative where the *uvrY *gene had been disrupted with a kanamycin resistance gene. Urine was collected through bladder punctuation at subsequent time intervals and the relative amount of *uvrY *mutant was determined. This showed that inactivation of the UvrY response regulator leads to a reduced fitness. In similar competitions in culture flasks with Luria Broth (LB) the *uvrY *mutant rather had a higher fitness than the wild type. When the competitions were done in flasks with human urine the *uvrY *mutant initially had a lower fitness. This was followed by a fluctuation in the level of mutant in the long-term culture, with a pattern that was specific for the individual urines that were tested. Addition of LB to the different urine competition cultures however clearly led to a consistently higher fitness of the *uvrY *mutant.

**Conclusion:**

This paper demonstrates that the BarA-UvrY two-component system is a determinant for virulence in a monkey cystitis model. The observed competition profiles strengthen our previous hypothesis that disruption of the BarA-UvrY two-component system impairs the ability of the bacteria to switch between different carbon sources. The urine in the bladder contains several different carbon sources and its composition changes over time. Inability to efficiently switch between the carbon sources may thus provide an explanation to the reduced fitness of the *uvrY *mutant in the cystitis model.

## Background

The rapid adjustment of bacteria to new conditions largely relies on two-component systems (TCS). In the prototypic model an environmental stimulus interacts with the N-terminus of a sensor protein, leading to autophosphorylation of a specific histidine residue using ATP. The sensor then acts as a kinase and transmits the phosphate group to a conserved aspartate residue on a cognate response regulator protein. This normally enables the regulator to control the transcription of a certain set of genes by sequence specific DNA binding.

Sequencing of the *Escherichia coli *genome has identified more than 30 TCS [1]. Four *Escherichia coli *sensor proteins, ArcB, EvgS, TorS and BarA, have two extra domains, D1 and H2, besides the H1 transmitter domain and are thus denoted as 'tripartite' [2-6]. The *Escherichia coli *tripartite sensor BarA (bacterial adaptive response) forms a TCS with the UvrY response regulator protein [7,8]. UvrY positively controls expression of the noncoding *csrB *and *csrC *RNAs [9-11], which bind the 6.8 kD CsrA protein and thus prevent it from interfering with ribosome loading of various target mRNAs [12]. The Csr (carbon storage regulation) system has been shown to have a major impact on regulation on carbon metabolism pathways [13]. Deletion of the *barA *or *uvrY *genes in *Escherichia coli*, as a consequence, has drastic effects on the ability of the bacteria to successfully grow in a competition depending on the carbon source of the medium [14]. Mutations in the Csr system in *Escherichia coli *and *Salmonella enterica *also cause other phenotypic effects, including changes in motility, adhesion and biofilm formation [10,15-19].

Deletions of orthologous systems in other bacteria; BarA-SirA in *Salmonella enterica*, ExpS-ExpA in *Erwinia carotovora*, BarA-VarA in *Vibrio cholerae *and GacS-GacA in *Pseudomonas spp*, clearly diminishes virulence of the bacteria [20,21]. The fact that this system is important for infections in a large spectrum of host organisms has also been corroborated by work demonstrating the necessity of the *Pseudomonas *GacS-GacA system for infections in either animals, nematodes, insects or plants [22]. The underlying mechanism behind this effect on virulence is far from clear. Several indirect effects have been reported, particularly on the formation of secreted molecules implied in the virulence of the bacteria [20], but the only verified direct target genes are functional homologues to the *csrB*/*csrC *regulatory RNAs as well as the genes encoding the HilA and HilC transcriptional regulators in *Salmonella *[10,23,24].

The nature of a physiological stimulus acting directly on the sensor is still elusive. Bile and intestinal short fatty acids have a clear effect on this two-component system in *Salmonella enterica *[25,26], although it is unclear if these effects are mediated *via *the sensor or not. Considering the fact that the system is activated in bacterial monocultures, we earlier proposed that a stimulus reflects the energy/growth status of the cell [14].

*Escherichia coli *is the causative agent in more than 80% of the uncomplicated human urinary tract infections [27]. *Macaque fascularis *monkeys express the digalactoside receptors to which the P fimbriated uropathogenic *Escherichia coli *strains bind and thus provide a suitable animal model system to study a cystitis [27]. Here we show that a mutant in the BarA-UvrY two-component system is less fit in a competition in such a cystitis model. We propose that this effect is at least partially mediated by a reduced ability to adapt the carbon metabolism in response to the varying carbon sources in urine.

## Results & discussion

### Monkey cystitis competition assay

**Figure 1:**
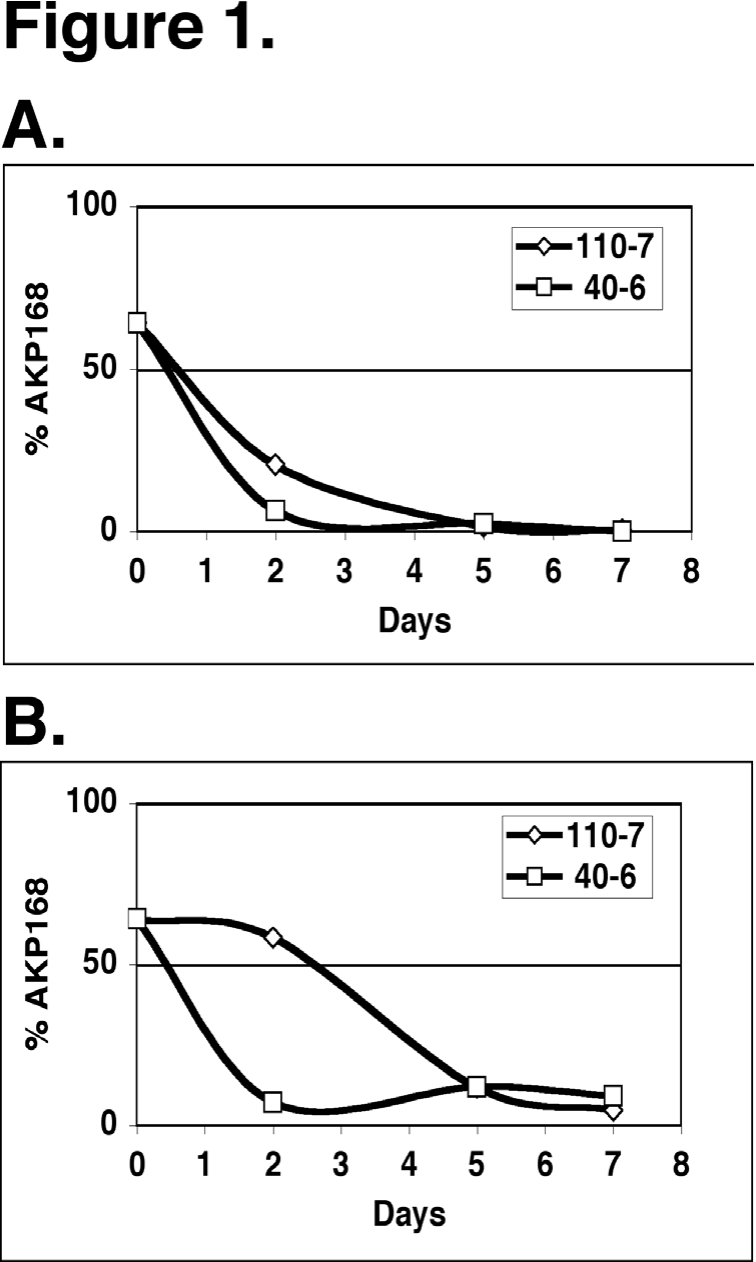
**The *uvrY *deletion mutant is outcompeted in the monkey bladder**. A 1:1 mixture of *DS17 *and *AKP168 *mutant was inoculated into the bladder of 2 individual monkeys (110-7 and 40-6) at day 0. Urine samples and vaginal smears were collected (day 2, 5 & 7). The percentage of the *AKP168 *mutant was determined in A. urine and B. vaginal smears.

Two female monkeys were inoculated in the bladder with a 1:1 mixture of the *DS17 *wild type and the *AKP168 uvrY *mutant. Urine samples were collected at day 2, 5, 7, 13 and 15 to determine the colony forming units (CFU) and the relative number of wild type and mutant bacteria. Both monkeys exhibited typical symptoms of an acute cystitis as measured by bacteruria and an increased level of leukocyte markers in the urine (Table 1). The infections were cleared naturally by day 13 from both animals (Table 1). Our analysis of the isolated samples clearly showed that the *uvrY *mutant *AKP168 *was rapidly lost in both monkeys, in comparison with the wild type *DS17 *(Figure 1A). On the fifth day of infection more than 95 % of the isolated bacteria were wild type. We also observed a similar take-over of the wild type strain in samples collected from the vagina in these monkeys (Figure 1B). This is expected, as urine continuously contaminates the vagina. We were however unable to perform additional infections as there were no more animals available for such a study.

### The BarA-UvrY two-component system controls the carbon utilization in the cell

We recently showed that the ability of a *uvrY *mutant to grow in a competition culture with the wild type depends on the growth medium, such that the mutant has a higher fitness than the wild type in gluconeogenic media, e.g. LB, but a lower fitness than the wild type in glycolytic media. These results led us to conclude that the BarA-UvrY TCS is important for the bacteria to efficiently switch between different carbon sources [14]. The switching ability is crucial in a glycolytic medium, as long-term growth will change the composition of the medium, with a gradual accumulation of secondary metabolites (e.g. acetate). Accumulated intermediates will in turn be metabolized when the primary metabolites are exhausted. When all carbon sources are depleted from the medium, lysed bacteria will be the only carbon source that can be used. The major carbon source in such lysates is amino acids [28]. In gluconeogenic media, based on amino acids or other small metabolites fuelling directly into the Krebs cycle, the composition of the medium will be relatively unchanged in a long-term culture, including the stage when amino acids from lysed bacteria will be the principal carbon source. Growth in such media is not directly aided by the BarA-UvrY TCS and mutants where the sensor or regulator has been disrupted are rather more fit than the wild type [14]. In support of our hypothesis, the BarA-UvrY TCS has been demonstrated to control transcription of the *csrB *and *csrC *noncoding RNAs [9,11,29], which via the CsrA protein regulate the expression of genes involved in gluconeogenis and glycolysis by inversely controlling the concentration of their mRNA at the post-transcriptional level [13]. In the case of csrB, UvrY has been proven to act directly on the csrB promoter [9].

### Growth of wild type and *uvrY *mutant strains in human urine

To test if the higher fitness of the wild type bacteria in the monkey cystitis model could be due to their ability to efficiently switch between different carbon sources present in urine we cultivated the bacteria in sterile filtered urine. The composition of urine varies between individual samples, but typically contains a mixture of amino acids, sugars and fatty acids as carbon sources [30,31].

**Figure 2:**
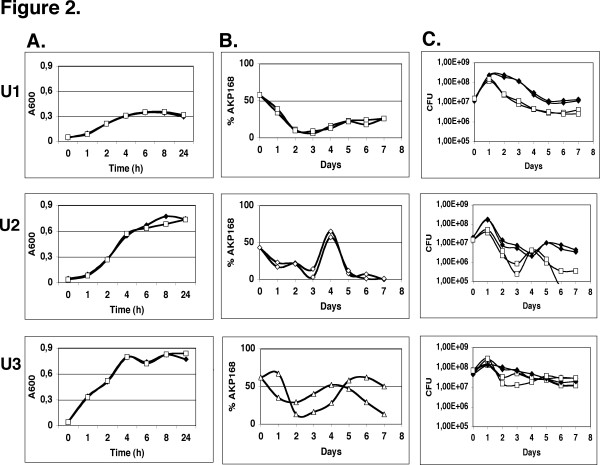
**Growth of individual strains and *In vitro *competition in human urine**. A. *DS17 *(filled diamonds) and *AKP168 *(squares) were grown separately *in vitro *in three different human urines (U1, U2 & U3). B. 1:1 mixture of *DS17 *and *AKP168 *mutant was grown in the three different human urines (U1, U2 & U3) and samples were taken at the indicated time points to determine the percentage of *AKP168 *mutant. C. The same competitions as in B. but plotted as CFU with *DS17 *depicted as filled diamonds and *AKP168 *as squares.

**Figure 3:**
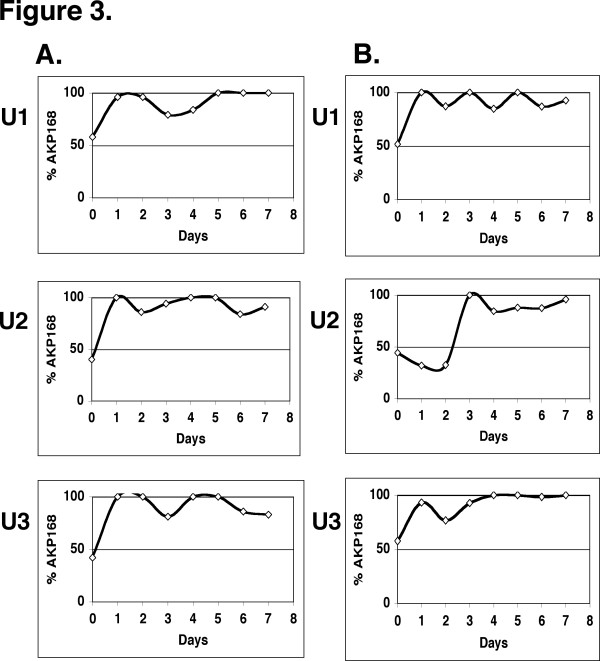
**In vitro competition in human urine complemented with LB**. A. Re-competition of *uvrY *mutants in LB. Three *AKP168 *individual mutant clones were isolated at day seven from the urine competitions (U1, U2 & U3) and competed against wild type *DS17 *in LB. Samples were taken at the indicated time points to determine the percentage of *AKP168 *mutant. B. The same experiment as in Figure 2B but the urine was complemented with LB (see Materials & Methods).

We first cultivated the *DS17 *and the *AKP168 *strains individually in urine freshly collected from three human subjects (U1, U2 & U3). The growth varied significantly between the individual urines but there was no significant difference in growth between the mutant and the wild type in any of the three urines (Figure 2A). We subsequently allowed the *DS17 *wild type and the *AKP168 *mutant to compete in these three human urines. Each competition culture was first split into two and the level of mutant was followed in each culture. In all six urine competitions the level of *uvrY *mutant decreased during the two first days, but this was followed by an increase in the level of *uvrY *mutant with a fluctuating pattern that was specific for each urine (Figure 2B and 2C). As urine is a complex medium consisting of a mixture of carbon sources, the initial decrease in the relative level of *uvrY *mutant may well be explained by its impaired ability to efficiently switch between gluconeogenic and glycolytic carbon sources. The competition profiles were markedly different between the urines but the same in the parallel cultures from the same urine (although with a slight time shift in U3). Our premise that the initial composition of the individual urine determines the competition profile agrees well with this reproducibility. The observed fluctuations after day 1–2 are thus unlikely to be explained by random processes or by spontaneous mutations in either the *AKP168 *or the wild type strain creating a growth advantage in stationary phase, GASP mutants [32,33]. From the results, it is more likely that each competition profile is decided by the initial composition of each individual urine. To exclude that the reduced fitness of the *uvrY *mutants in the urine cultures was the result of additional weakening mutations, we demonstrated that mutants taken at day 7 from the different urine competitions could successfully outcompete the wild type strain in a competition in LB (Figure 3A).

The increase in the level of mutant after the initial decrease may be explained by the massive lysis of bacteria after the entry into stationary phase. Lysis will provide an environment with an excess of gluconeogenic carbon sources, where it is known that a *uvrY *mutant has a growth advantage [14].

We also repeated the competition in the same urines (U1, U2 & U3) complemented with LB. In these competitions where there is a constant excess of gluconeogenic carbon sources in the medium, the *uvrY *mutant rapidly outcompeted the wild type (Figure 3B), similar to what is observed in LB alone (Figure 3A). Thus, there seems to be no dominant factor in urine that would explain the lowered fitness of the *uvrY *mutant.

Although our model system cannot mimic the exact conditions in the bladder, such as the continuous addition of fresh urine, the data suggest that a *uvrY *mutant has an impaired ability to efficiently metabolize the different nutrient components in the urine. This also emphasizes that an optimal growth rate is critical for bacteria during an infection. In analogy with our results, it is interesting to note that long-term growth of *Pseudomonas *strains in gluconeogenic media selects for strains with inactivating mutations in the orthologous GacS-GacA system, unlike when the same strains are tested in an infection model (*Arabidopsis*) [34]. It would be interesting to test what role the described metabolic switches plays in the other bacterial species where it is known that the BarA-UvrY two-component system family affects virulence.

We cannot exclude that other factors under the control of this TCS contributes to the lowered fitness of the *uvrY *mutant observed in the *in vivo *cystitis model. Earlier studies have demonstrated effects of the BarA-UvrY TCS and the downstream Csr system on adhesion, aggregation and biofilm formation [10,15-19], all of which may play a role in the persistence of bacteria in the bladder in concert with the ability to utilize and switch between glycolytic and gluconeogenic carbon sources.

## Conclusion

This paper demonstrates that the BarA-UvrY system is a determinant for virulence in a monkey cystitis model. Based on different competition profiles we propose that the BarA-UvrY TCS is important for the bacteria to efficiently switch between different carbon sources present in urine. This would provide a plausible explanation to the reduced fitness of the *uvrY *mutant in the cystitis model as urine consists of several different carbon sources and as the prevalent carbon sources will change over time. This also agrees well with the observed higher fitness of the *uvrY *mutant in competitions in purely gluconeogenic media as the *uvrY *mutant is predisposed for gluconeogenesis. In conclusion, the ability to grow quickly and benefit from the available carbon and energy sources is crucial for virulence. During infection there may be different carbon sources available and we believe that the ability to know when to metabolize them is in part conferred by the BarA-UvrY two-component system.

## Methods

### Monkey cystitis competition model

Two adult female *Macaque fascularis *monkeys were infected by inoculation of a bacterial suspension (1 ml of 10^8 ^bacteria/ml), containing equal amounts of *DS17 *wild type and the isogenic *uvrY *mutant *AKP168*, into the bladder using a catheter (the bladder void volume is approximately 150 ml). The monkeys had not been exposed to the *DS17 *strain earlier. Bacteria had been taken from fresh colony forming agar (CFA) plates (10 g Casamino acids, 1.5 g yeast extract, 50 mg MgSO_4_, 5 mg MnCl, 15 g Bacto agar per liter) and resuspended in phosphate buffered saline (PBS). Inocula were directly analyzed on selective plates to assure that the level of mutant was close to 50 %. Samples from monkeys were collected by suprapubic bladder aspiration, using a syringe with vacutainer, at the time points indicated until two successive negative cultures were obtained (day 13 and day 15; Table 1). The animals were then considered healthy and removed from the study. At 20–30 min before bladder aspiration, monkeys were hydrated with approximately 50 ml of lukewarm saline administered subcutaneously in the neck for optimal diuresis. All experiments were done under ketamine and midazolam anesthesia. Vaginal smears were collected with cotton swabs. No *DS17 *bacteria could be isolated from the feces of the monkeys before or after the infection. Monkeys were kept in the animal facility at the Swedish Institute for Infectious Disease Control (SMI) in 80 × 70 × 170 cm (width, depth, and height) cages, two in each, and were handled by professional personnel according to SMI regulations. Experiments were pre-approved by the local ethics committee in Stockholm.

### *In vitro *growth of strains

In this work we employed the uropathogenic *Escherichia coli *strain *DS17 *[35] and the *AKP168 *derivative [14], with the *uvrY *gene disrupted by a kanamycin resistance cassette. The *AKP168 *mutant exhibited no measurable difference in growth relative the parental *DS17 *strain in a range of different media, as measured by optical density and CFU [14].

Growth was normally conducted in a 20 ml volume in 100 ml Erlenmeyer glass flasks at 37°C in a shaker with 200 rpm. Urine collected from healthy human male donors was centrifuged at 4000 rpm and sterile filtered through a 0.45 μM CA-filter (Corning Inc., NY). Urine-LB was prepared by mixing four parts of sterile filtered urine with one part of 5 × LB (50 g Bacto tryptone, 25 g Bacto yeast extract and 50 g NaCl per liter).

In growth curve experiments each strain was grown as an over night culture in LB (10 g Bacto tryptone, 5 g Bacto yeast extract, 10 NaCl per liter) started directly from a -80°C vial), washed twice in PBS, inoculated 1:100 in 20 ml urine. 1 ml samples were withdrawn and the optical density was measured at 600 nm at the indicated time points with a spectrophotometer.

In competition experiments each strain was grown as an over night culture in LB (started directly from a -80°C vial), washed twice in PBS, inoculated 1:100 in urine, urine-LB, or plain LB, and grown as an individual culture to A_600 _= 0.15–0.30. Subsequently, cultures were mixed 1:1 to a 20 ml volume. Samples of 100 μl were withdrawn at the indicated time intervals and subjected to appropriate dilution in PBS. Dilutions were added to Luria-Bertani plates, with or without kanamycin (10 μg/ml), together with 5 to 6 glass beads (6 mm diameter) (Stern Walter Inc, NY, USA). Plates were gently agitated for 20 seconds to evenly spread the bacteria over the plate, after which the beads were removed. The relative level of mutant was calculated by dividing the number of colonies growing on the selective plate with the number of colonies growing on the non-selective plate.

## Authors' contributions

HT, AKP and KJ carried out the experimental work. RM provided expertise for the primate experiments. All authors have been involved in the design and planning of the experiments and the drafting of the manuscript. OM coordinated the study. All authors read and approved the final manuscript.

## Figures and Tables

**Table 1:** Leukocyte markers and CFU

Day	Leukocytes^a^		CFU in urine (CFU/ml)	
	
	110-7^b^	40-6^b^	110-7^b^	40-6^b^
2	1–2	2–3	9.5 × 10^4^	4.6 × 10^5^
5	3	2–3	3.2 × 10^5^	2.7 × 10^5^
7	1–2	1	5 × 10^2^	2.5 × 10^4^
13	0–1	0–1	0	0
15	0	0	0	0

^a^Presence of leukocytes was determined by the ECUR-dip stick test (Boehringer-Mannheim). Color changes of grades 1, 2 and 3 correspond to 10–25, approximately 75, and approximately 500 granulocytes/ml, respectively.

^b^Identification number for the individual monkeys.
